# Supplementary material for: Metamaterial bricks and quantization of meta-surfaces
Source: Nat Commun. 2017 Feb 27;8:14608. doi: 10.1038/ncomms14608 (PMC5333366; doi:10.1038/ncomms14608)
Supplement: Supplementary Information — Supplementary Figures, Supplementary Notes and Supplementary Table [file ncomms14608-s1.pdf]

Supplementary Note 1. Details of the wavelet-based compression

A widely used compression algorithm is the JPEG standard of image encoding, which is based on discrete cosine transforms (DCTs). We discard this compression procedure as it cannot capture abrupt changes in φ_{ij} that are already present in a focusing meta-surface. In this study we used a compression algorithm based on the discrete wavelet transform (DWT). Based on waveforms with an average value of zero (*i.e., parent wavelets*), the DWT is typically used in image processing to separate the fine details in an image. This is achieved by decomposing the image into a superposition of shifted and scaled representations of the original parent wavelet, which are mutually orthogonal. The procedure generates not a single matrix of coefficients, as in the DCT case, but a hierarchical tree of matrices where the spatial resolution doubles at each step (Supplementary Fig. 1). These matrices together form the transform φ_K^{DWT}, where K is the level of decomposition. In this work, we used the Haar wavelet $H(x)$, which corresponds to a square wave in the interval of definition.

Once an image is decomposed, compression can be achieved by decimating the coefficients of the wavelets below a certain threshold value δ. The inverse transform $\tilde{\varphi}_{ij}$ is then computed, which contains a number of unique phases dependant on the threshold chosen (*i.e.* on the compression level for the specific image). This is a lossy process, as information is reduced by the thresholding step: a lossless approach is detailed later.

It is worth noting that, since the coefficients depend on the parent wavelet chosen (*i.e.* on the base used), we tried also the Shannon wavelet $S(x)$, *i.e.* the function $2(\sin 2\pi x - \cos \pi x)/(\pi - 2\pi x)$ in the λ_0-wide interval of definition, yielding to similar results.

Supplementary Note 2. Discussion on phase quantization

As mentioned in the main text, various authors consider an equally spaced *M*-subdivision interval $[0, 2\pi)$ and then compute a histogram of φ_{ij} using *M* bins. This results in a simple AD conversion step: substitute each analogue phase value with the corresponding bin value, such that $\tilde{\varphi}_{ij}^{(M)} = \varphi_m;$ here, $\varphi_m - \frac{\pi}{M} < \varphi_{ij} < \varphi_m + \frac{\pi}{M}$, with $i, j = 1 \dots N$, and $m = 1 \dots M$.

To decide on the optimal number of unique phases, *i.e.* quanta, we define the error function:

$$\varphi_{\mathrm{E}}^{(N)}(M) = \sqrt{\sum_{j=1}^{N} \sum_{i=1}^{N} \frac{1}{N^2} (\varphi_{ij} - \tilde{\varphi}_{ij}^{(M)})^2} \qquad (1)$$

where $\varphi_{\mathrm{E}}^{(N)}(M)$ represents the error in reproducing the analogue phase distribution φ_{ij} for a N×N grid with *M* available phases.

Supplementary Fig. 2a shows the value of $\varphi_{\mathrm{E}}^{(16)}(M)$ for a 16×16 grid emulating a focused beam with a focal point F_0 varying from $3\lambda_0$ and $47\lambda_0$ (26 mm and 400 mm at 40 kHz) along the axis; here, the number of discrete phases varies from $M = 2$ to $M = 50$. A simple piston-like model was implemented to produce a focused beam. A very small relative reduction in φ_{E} is noted when using more than 16 phases, and the same effect can be observed in the pressure distribution in the focal region (Supplementary Fig. 2b): a 4-

bit quantisation (16 unique phases) yields to a maximum approximation error of 0.1 dB. Supplementary Fig. 2b also shows that, if a 2-bit (4 unique phases) or a 3-bit (8 unique phases) quantization is used, the sound pressure level (SPL) maintains the same trend as the one generated by an analogue phase distribution, with differences as large as 1 and 3 dB, respectively. As a result of this analysis, we have selected a hexadecimal phase distribution for the metamaterial bricks in the main text.

Supplementary Note 3. Additional information on the brick design

The brick design described in the main text was selected among other possible ones, as shown in Supplementary Fig. 3, in order to maximise the robustness of the assembly process and to minimise the energy loss across the single layer, thus facilitating stacking.

Supplementary Table 1 reports in brief the key characteristics of the bricks used in the main text. Particularly important for stacking is the transmission coefficient, which was found to be 0.98 ± 0.02. Note that the thickness of the brick walls was fixed to $\lambda_0/40$, while the bar thickness is $\lambda_0/20$; the radius of the bar fillets is such that $\rho_r = \min(b_l, \lambda_0/10)$.

Supplementary Note 4. Realisation of a focusing meta-surface

Supplementary Fig. 4 reports the realisation of a focusing meta-surface with a focal point at $F_0 = (0{,}0{,}100)$. Here, a uniform 4-bit quantization of the phase domain is compared both numerically and experimentally with a non-uniform 3-bit quantization.

Supplementary Note 5. Physical realisation of the wavelet decomposition

A wavelet transform is a signal decomposition technique that can achieve both lossless and lossy signal compression. In the main text and in Supplementary Fig. 4 we used this decomposition to achieve a lossy compression. There is, however a lossless alternative, conceptually illustrated in Fig 1c (of the main text).

As discussed in Section S1, we used the Haar function, $H(x)$, as a parent wavelet: at the first level of the wavelet hierarchy, this is a signal of amplitude 1, when $x \in (0, \lambda_0/2)$, and of amplitude -1, when $x \in (\lambda_0/2, \lambda_0)$. As shown in Supplementary Fig. 1, the original 4-bit image is decomposed into:

$$\varphi_{ij} = \{0\} \times H_{\lambda_0} + \{1\} \times H_{\lambda_0} + \{2\} \times H_{\lambda_0} + \{3\} \times H_{\lambda_0} \qquad (2)$$

where $\{0\}, \{1\}, \{2\}$ and $\{3\}$ are the matrices of the coefficients in Supplementary Fig. 1, and H_{λ_0} is the spatial representation of the Haar function.

Since phases are additive, it is possible to obtain the sum in supplementary equation (2) in an experimental way by stacking four different meta-surfaces: each will represent one of the addends in supplementary equation (2).

Each coefficient of the wavelet transform then applies to a 2×2 part of the grid. In terms of bricks, this means that in each 2×2 cell, the first two bricks will have a phase given by the corresponding coefficient of the wavelet transform, while the other two will have a phase opposite. Since phases are modular to 2π, however, a negative phase $-\phi_0$ will need to be reported as $2\pi - \phi_0$ and the same applies to the coefficients between $[-2\pi, 8\pi]$ reported in Supplementary Fig. 1.

In this example, we exploited both modularity and additivity of phases by identifying the Haar function with a brick of the same spatial resolution. In this way, the coefficients of the first hierarchical level become the phase that needs to be represented by a brick and each matrix of coefficients can be seen as a meta-surface with a reduced spatial resolution. This method, detailed in Fig. 1c of the main text, is technically lossless. An example of successful application can be found in Supplementary Fig. 5, where we realise a focusing meta-surface (previously realized in Supplementary Fig. 4) with by stacking the 4 meta-surfaces corresponding to the first level of the wavelet hierarchy. We observed the focus at ~ 80 mm, with a spot size in line with the Rayleigh principle. Crucially, this method exploits stacking to represent φ_{ij} using 3.5 bits and a spatial resolution λ_0.

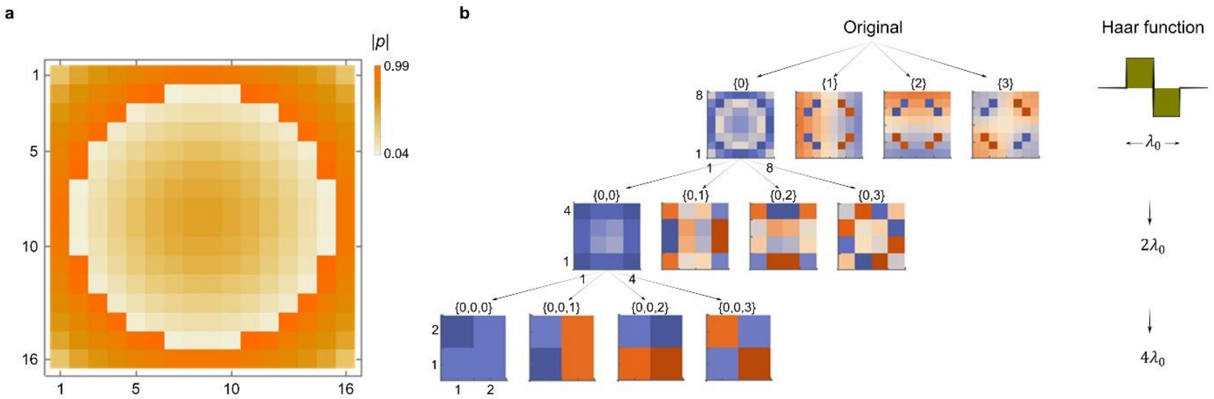

Supplementary Figure 1 | Example of discrete wavelet transform. **a**, Original phase distribution φ_{ij} to form a focus at (0,0,100) mm where the 2π-range has been normalised to 1. Note here the size of each pixel in the 16×16 grid is $\lambda_0/2$. **b**, Hierarchical representation of the DWT using the Haar wavelet. Here, the coefficients in the hierarchical representation cover the range $[-1, 4]$, which is much larger than the original [0,1].

	#1	#2	#3	#4	#5	#6	#7	#8	#9	#10	#11	#12	#13	#14	#15
**Target phase angles**	22.5°	45°	67.5°	90°	112.5°	135°	157.5°	180°	202.5°	225°	247.5°	270°	292.5	315°	337.5°
**Actual phase angles**	23.3°	47.5°	67.4°	89.7°	115.6°	134.4°	159.3°	177.3°	204.8°	226.2°	246.4°	271.4°	295.3°	315°	335.3°
**Transmission coefficient (magnitude)**	0.987	0.999	0.973	1.0	0.999	0.993	0.999	0.997	0.963	1.0	0.977	1.0	0.999	1.0	1.0
**Bar length, b_ℓ / λ_0**	0.062	0.092	0.112	0.132	0.152	0.162	0.171	0.191	0.221	0.241	0.251	0.271	0.281	0.301	0.321
**Inter-bar spacing, b_s / λ_0**	0.216	0.212	0.207	0.189	0.161	0.166	0.171	0.134	0.257	0.234	0.230	0.207	0.203	0.175	0.152
**Fillet radius, ρ_r / λ_0**	0.062	0.092	0.1	0.1	0.1	0.1	0.1	0.1	0.1	0.1	0.1	0.1	0.1	0.1	0.1

Supplementary Table 1 | Key characteristics of the metamaterial bricks used in this study.

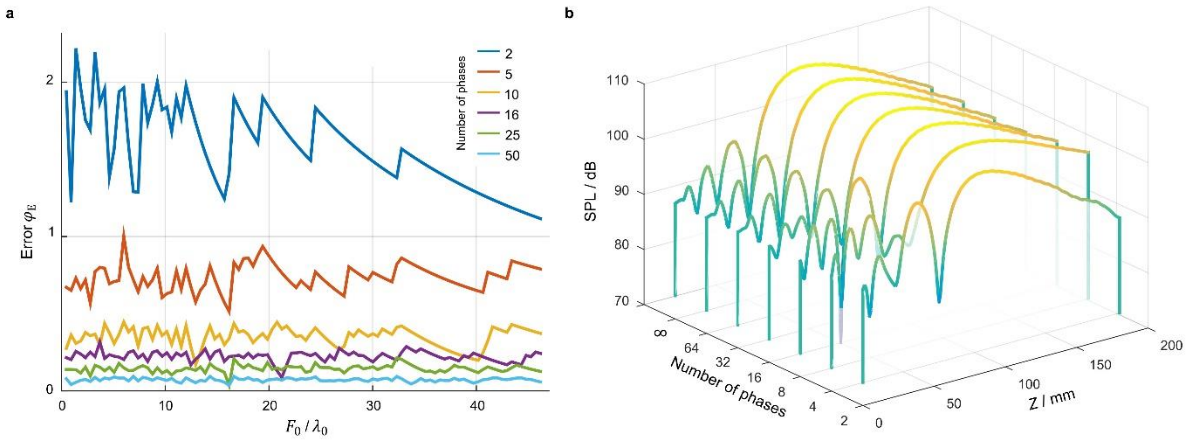

Supplementary Figure 2 | Effect of the phase quantization on a 16x16 grid. a, Error in reproducing the analogue phase distribution for varying focal distances along the axis. **b,** Sound pressure level (SPL) along the axis for different numbers of M uniformly distributed phases.

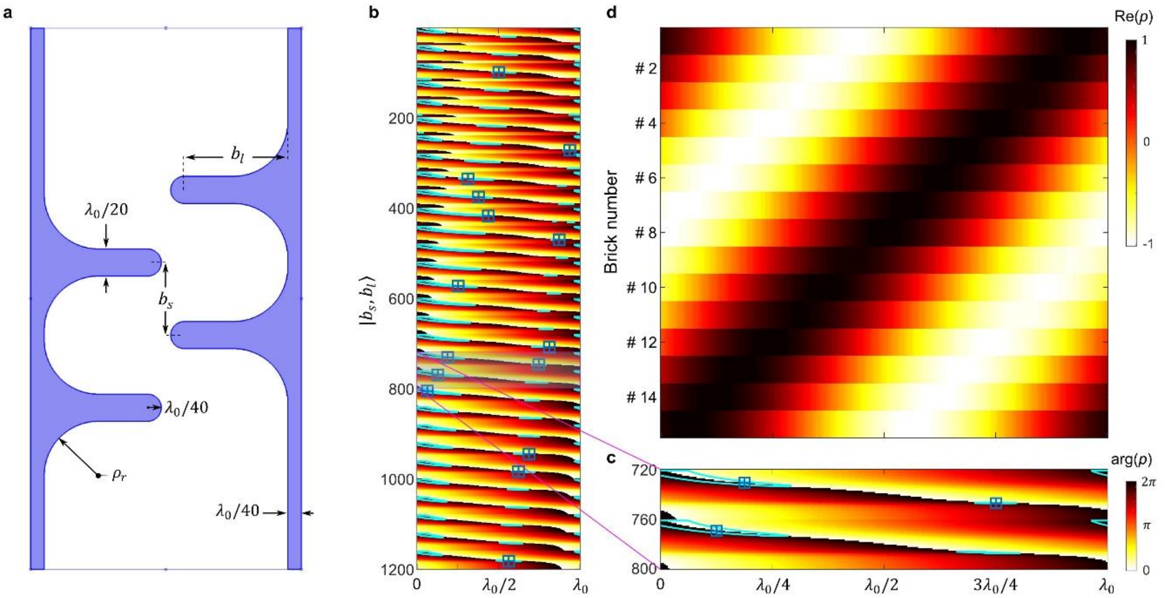

Supplementary Figure 3 | Parametric sweep study on $|b_s, b_\ell\rangle$ and tabulation of the selected data. a, Geometrical details on the design of the bricks (see also Supplementary Table 1). **b**, Transmitted phase map through a brick build with 1200 possible combinations of varying bar length b_ℓ (30 points) and inter-bar spacing b_s (40 points). The overlaid contour corresponds to the pressure field $Re(p)$ at a constant level of $|p| = 0.95$ – this is the threshold used in this study. The symbols represent the 15 selected parameters with a phase delay swiping uniformly the interval $[0,2\pi)$. **c**, Zoom of the selected region in figure **b**; here, *e.g.* the index interval (720:760) corresponds to $\{b_s^{(1:30)}, b_\ell^{18}\}$. **d**, Pressure field map showing a clear constant phase shift over a wavelength λ_0 for 15 selected bricks.

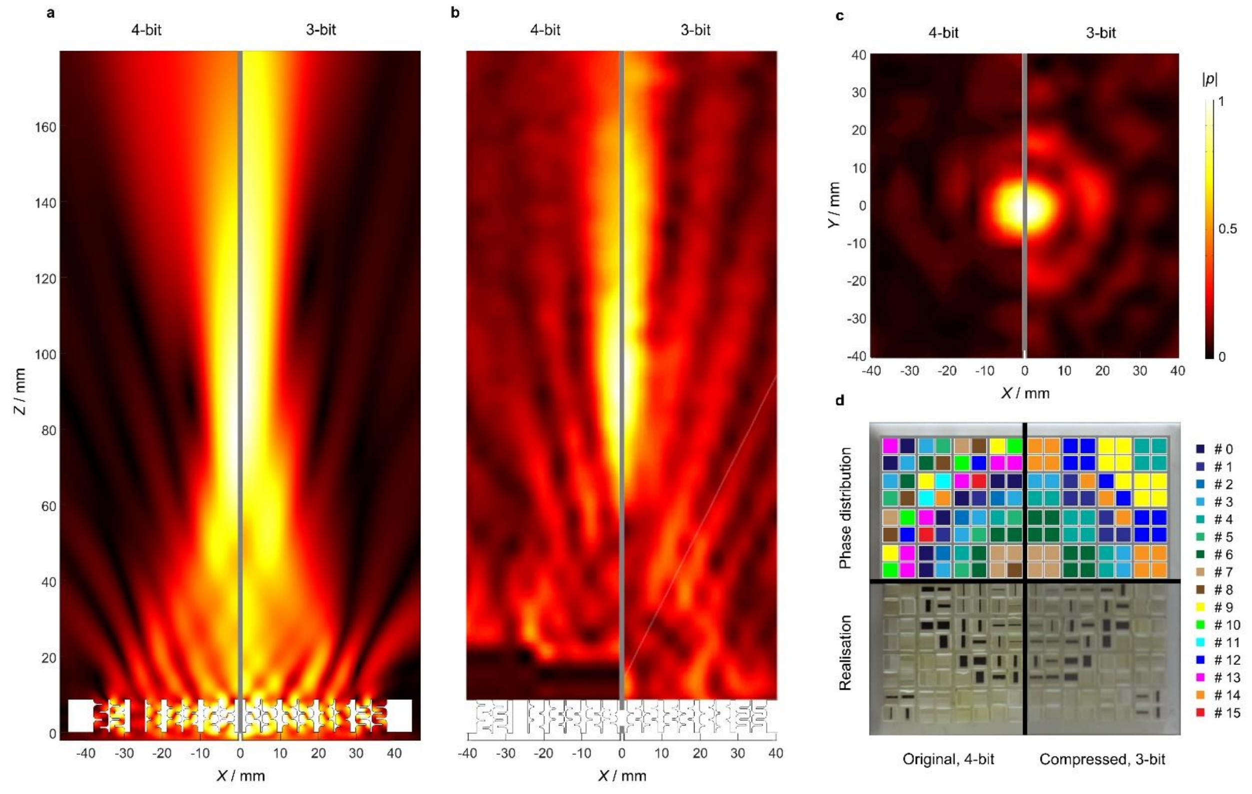

Supplementary Figure 4 | Effect of the different phase quantization levels for a focusing surface. **a**, Simulations and **b**, measurements showing the pressure field maps at different bit-rates. Here, the pressure field is probed in the vertical plane $Y = 0$. **c**, Experimental pressure field maps in the horizontal plane at $Z = 100$ mm. **d,** Schematic design of the two meta-surfaces and their practical realization.

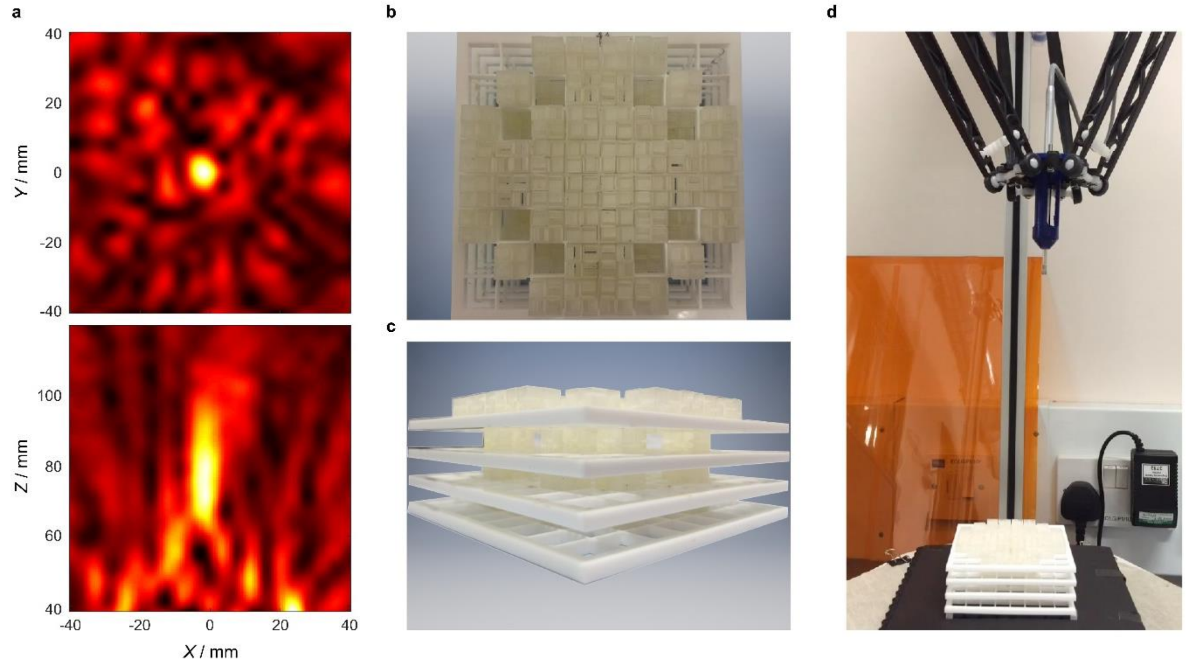

Supplementary Figure 5 | Physical realization of the wavelet decomposition by stacking. a, Measurements showing the pressure field maps in planes at $Z \approx 80$ mm (top panel) and $Y = 0$ (bottom panel). **b-c**, Photographs of the fabricated stack (top and side views, respectively). **d**, Experimental setup used for microphone measurements.
